# Microglial Sestrin2 alleviates depressive-like behaviors and cognitive impairment in a YTHDF1-dependent manner

**DOI:** 10.3389/fnins.2026.1894304

**Published:** 2026-07-08

**Authors:** Ming Sun, Jiaming Shen, Rongrong Huang, Lixiang Yang, Zhenyu Fan

**Affiliations:** 1 Department of Ultrasound, Affiliated Hospital of Nantong University, Nantong, China; 2 Department of Pharmacy, Huai’an Maternal and Child Health Hospital, Huaian, China; 3 Clinical Research Center, Nantong Research Institute of Traditional Chinese Medicine, Nantong Hospital of Traditional Chinese Medicine, Nantong Hospital Affiliated to Nanjing University of Chinese Medicine, Nantong, China; 4 Department of Neurosurgery, The Affiliated Wuxi People’s Hospital of Nanjing Medical University, Wuxi People’s Hospital, Wuxi Medical Center, Nanjing Medical University, Wuxi, Jiangsu, China; 5 Department of Pharmacy, Affiliated Hospital of Nantong University, Nantong, China

**Keywords:** cognitive impairment, depression, microglia, Sestrin2, YTHDF1

## Abstract

**Objective:**

This study aims to investigate the role of microglial Sestrin2 in chronic unpredictable stress (CUS)-induced depressive-like behaviors and cognitive impairment in mice, and to explore the upstream molecular mechanism underlying the abnormal expression of microglial Sestrin2.

**Methods:**

Microglia-specific overexpression of Sestrin2 was achieved by injecting adeno-associated virus (AAV) into the CUS mouse hippocampus. Depressive-like behaviors were assessed using sucrose preference, tail suspension, and forced swim tests. Cognitive function was evaluated by the Morris water maze. Levels of IL-1β and IL-6 in the hippocampus and cell supernatants were measured by ELISA. BV2 microglial cells were used for *in vitro* mechanistic studies. YTHDF1 siRNA and overexpressive lentivirus were used to regulate YTHDF1 expression in vitro. RNA immunoprecipitation was performed to demonstrate the physical interaction between YTHDF1 and Sestrin2 mRNA.

**Results:**

Sestrin2 expression was significantly reduced in the hippocampus of CUS mice. Overexpression of Sestrin2 specifically in microglia ameliorated CUS-induced depressive-like behaviors, cognitive impairment, and inflammatory levels. YTHDF1 expression was also reduced in the CUS hippocampus. Mechanistically, YTHDF1 bound to Sestrin2 mRNA and knockdown of YTHDF1 decreased Sestrin2 expression. Molecular biology prediction results showed that positions 1943 and 2,114 of Sestrin2 mRNA are high-confidence N^6^-methyladenosine (m^6^A) modification sites. Mutation of the 2,114 site on Sestrin2 mRNA inhibited the effect of YTHDF1 on 3Flag expression. Furthermore, YTHDF1 knockdown promoted IL-1β and IL-6 production in BV2 cells, which was reversed by Sestrin2 overexpression.

**Conclusion:**

Microglial Sestrin2 alleviates depressive-like behaviors, cognitive impairment and neuroinflammation. YTHDF1 regulates Sestrin2 expression via an m^6^A-dependent mechanism, and the YTHDF1-Sestrin2 axis may represent a novel therapeutic target for major depressive disorder.

## 1 Introduction

Major depressive disorder (MDD) represents one of the most prevalent and disabling mental health conditions globally, affecting an estimated 300 million people across diverse populations (Yuan et al., 2023; Bai et al., 2024). Clinically, the disorder is defined not only by persistent low mood and diminished interest but also by a spectrum of cognitive disturbances that frequently compromise daily functioning (Guan et al., 2025; Miao et al., 2025; Yan et al., 2024). Emerging evidence suggests that such cognitive impairments are not merely epiphenomena of emotional distress but rather integral components of the illness (Zhang et al., 2025). These deficits, which range from attentional lapses to executive dysfunction, often persist beyond the resolution of affective symptoms (Pearson et al., 2023; Pan et al., 2019). The persistence of these cognitive deficits poses a substantial barrier to functional recovery, underscoring the need to elucidate the underlying neurobiological substrates linking depressive pathology with cognitive decline.

Chronic inflammation has gained considerable attention as a contributing factor in the pathogenesis of MDD (Ahmed Abbasi et al., 2025). Elevated circulating levels of pro-inflammatory mediators such as interleukin-1β (IL-1β), interleukin-6 (IL-6), and tumor necrosis factor-*α* (TNF-α) have been consistently documented in MDD patients, and these immunological alterations have been associated with both the severity of depressive symptomatology and the degree of cognitive compromise (Huang et al., 2022; Solomon et al., 2025). The hippocampus is a key brain structure involved in learning and memory processes and exhibits marked sensitivity to inflammatory insults (Huang et al., 2023). Neuroimaging studies have revealed reduced hippocampal volume in individuals with depression, with the extent of structural alterations correlating with illness duration and episode frequency (Sun et al., 2023; Schmaal et al., 2016). Microglia, the principal immune-competent cells of the central nervous system, serve as key mediators of inflammatory responses within the brain (Harackiewicz and Grembecka, 2024; Liu et al., 2025). In depression, microglia secrete pro-inflammatory cytokines that subsequently interfere with neuronal function, suppress adult neurogenesis, and impair synaptic plasticity, processing that are critically important for cognitive function (Wu and Zhang, 2023; Li et al., 2025; Yang et al., 2024). Although the role of microglial inflammation in the hippocampus in depression has been elucidated to some extent, the development of therapeutic targets capable of addressing the multidimensional symptomatology of depression remains a considerable challenge.

Sestrin2 is a conserved stress-inducible protein that integrates metabolic and inflammatory signaling. Recent evidence highlights the anti-inflammatory role of Sestrin2, including inhibition of NLRP3 inflammasome activation and reduction of pro-inflammatory cytokines in macrophages and microglia (Kim et al., 2016). Multiple clinical studies have indicated that Sestrin2 may be an important determinant in the pathophysiology and etiology of MDD and its subtypes, while also contributing to the assessment of clinical severity and the identification of new therapeutic targets (Aslan et al., 2023; Qian et al., 2023). Animal experiments have shown that overexpression of attenuates depressive-like behaviors and neuroinflammation in chronic unpredictable stress (CUS) mice through inhibiting ferroptosis (Ma et al., 2024). However, the cellular and molecular mechanisms underlying the antidepressant effects of Sestrin2 remain poorly understood.

In this study, we found that Sestrin2 expression was significantly reduced in the hippocampus of CUS mice. Overexpression of Sestrin2 specifically in microglia suppressed depressive-like behaviors and cognitive impairment in these mice. Subsequent experimental results indicated that the reduction in microglial Sestrin2 expression was mediated by YTHDF1 depending on the N^6^-methyladenosine (m^6^A)-modified site. Targeting the YTHDF1-Sestrin2 axis to modulate microglial inflammation may represent a novel potential strategy for the future treatment of MDD.

## 2 Materials and methods

### 2.1 Establishment of animal model

Adult male C57BL/6 J mice (6–8 weeks old) were exposed to a 5-week CUS treatment. To maintain unpredictability, two stressors were administered in a random order each day. These stressors comprised: food or water withdrawal for 24 h; cages tilted at 45° for 12 h; tail clamping for 5 min; swimming in cold water for 6 min; confinement for 2 h in a ventilated 50 mL centrifuge tube; damp bedding for 24 h; 12 h light/dark cycle reversal for 24 h; and horizontal cage shaking for 30 min. Control mice were kept under standard housing conditions with a 12 h light/dark cycle and unrestricted access to food and water. Behavioral assessments were performed 24 h after the final stress session. All experimental procedures were approved by the Animal Ethics Committee of Nantong University.

### 2.2 Behavior tests

#### 2.2.1 Sucrose preference test

Mice were singly housed and acclimated to two bottles for 2 days. On day 1, both bottles contained water; on day 2, one bottle was replaced with 1% sucrose solution. Bottles were switched every 12 h. On test day, mice were water/food deprived for 4 h, then given access to one bottle of 1% sucrose and one bottle of water for 12 h. Sucrose preference (%) = [sucrose intake/(sucrose intake + water intake)] × 100%.

#### 2.2.2 Tail suspension test

Mice were suspended by the tail from a horizontal bar (50 cm above the floor) using adhesive tape placed 1 cm from the tip of the tail. Each mouse was tested once for 6 min. The total duration of immobility (passive hanging without limb movements) was recorded during the last 4 min. Immobility time was analyzed as a measure of behavioral despair.

#### 2.2.3 Forced swim test

Mice were individually placed into a transparent cylinder (height 25 cm, diameter 15 cm) filled with water (23–25 °C) to a depth of 15 cm, ensuring mice could not touch the bottom. Each test session lasted 6 min. The total duration of immobility (floating without struggling, only minimal movements necessary to keep the head above water) was recorded during the last 4 min. Immobility time was quantified as a measure of behavioral despair.

#### 2.2.4 Morris water maze test

The experiment consisted of three phases: first, a one-day visible platform training session; second, five consecutive days of hidden platform trials (four trials per day); and finally, a probe trial on the last day, during which the platform was removed. Mouse movement trajectories were recorded using a video tracking system, and the acquired images and data were subsequently analyzed.

### 2.3 Adeno-associated virus injection

Mice were anesthetized by intraperitoneal injection of tribromoethanol and secured in a stereotaxic frame. Either control or Sestrin2-overexpressing eGFP-tagged AAV (CD68 promoter, 1.2 μL, 10^12^ viral genomes/μL, HANBIO, China) was slowly infused into the hippocampus. Stereotaxic coordinates relative to bregma were: AP–2.06 mm, ML ± 1.5 mm, and DV–2.0 mm from the skull surface. One week after AAV injection, mice were subjected to CUS establishment protocol.

### 2.4 Cell culture

BV2 cells were purchased from Procell (Wuhan, China) and cultured in Dulbecco’s modification of Eagle’s medium (DMEM, C11995500, Gibco) with fetal bovine serum (FBS, Z7186FBS, Zeta Life) and penicillin- streptomycin (PS, C125C5, NCM Biotech) at 37°C under the condition of 5% CO2. BV2 cells were treated with LPS (100 ng/mL) for 24 h.

### 2.5 SiRNA and plasmid transfection

BV2 cells were seeded into 24-well plates at a density of 2 × 10^5^ cells per well and cultured for 24 h prior to transfection. Cells were then transfected with either YTHDF1 siRNA or control siRNA, using the GP-transfect-Mate transfection reagent (G04008, GenePharma, China), according to the manufacturer’s protocol. The transfection complex was maintained for 48 h before subsequent analysis. All siRNA molecules used in the experiments were designed and synthesized by GenePharma. YTHDF1 siRNA sequence was 5’-TGACAGTAACTCTGTTGGAAATGCCCAAC-3′. Transfection of the 3 × Flag-tagged plasmid was performed similarly to that of siRNA.

### 2.6 Lentivirus infection

BV2 cells were seeded in 24-well plates at 2 × 10^5^ cells per well and transduced with Sestrin2-overexpressing lentivirus or control lentivirus (GenePharma, China) at an MOI of 10 in the presence of polybrene (5 μg/mL). After 24 h, the virus-containing medium was replaced with fresh complete medium, and cells were cultured for an additional 48 h before further analysis.

### 2.7 Measurement of inflammatory factors

Mouse hippocampal tissues were homogenized in ice-cold PBS and were centrifuged at 3,000 rpm for 5 min at 4 °C, and supernatants were collected. Total protein concentration was determined using a BCA kit (P0009, Beyotime, China). For BV2 cell culture supernatants, cell culture media were collected and centrifuged at 1,000 g for 5 min at 4 °C to remove cell debris. The IL-1β and IL-6 level was measured by commercial ELISA kits (PI301 and PI326, Beyotime, China). All procedures were performed according to the manufacturer’s instructions. A standard curve was generated by plotting the absorbance at 450 nm against the concentrations of the standards, and the points were connected using a smooth line. The concentration of IL-1β and IL-6 in each sample was then interpolated from this standard curve based on its respective absorbance value.

### 2.8 Real-time PCR

Total RNA was extracted from cells and tissues using RNAeasy Isolation Reagent (RC112-01, Vazyme, China) according to the manufacturer’s protocol. RNA concentration was measured with a NanoDrop2000, and 200 ng of RNA was reverse-transcribed into cDNA using HiScript III RT SuperMix (R323-01, Vazyme, China). RT-PCR was performed using SYBR qPCR Master Mix (Q711-02, Vazyme, China) on a Step One Plus Real-Time PCR System. GAPDH served as the internal control, and relative expression levels were calculated as fold changes relative to the control group. Primer sequences are listed in Table 1.

**Table 1:** The sequence information of primers used in this study.

Primer	Sequence (5′–3′)
Sestrin2 forward primer	TCCGAGTGCCATTCCGAGAT
Sestrin2 reverse primer	TCCGGGTGTAGACCCATCAC
GAPDH forward primer	AGGTCGGTGTGAACGGATTTG
GAPDH reverse primer	TGTAGACCATGTAGTTGAGGTCA

### 2.9 RNA immunoprecipitation

BV2 cells were lysed in complete RIP Lysis Buffer containing protease and RNase inhibitors. Lysates were incubated with magnetic Protein A/G beads conjugated to 5 μg of anti-YTHDF1 (17479-1-AP, Proteintech, China), anti-YTHDC2 (27779-1-AP, Proteintech, China), or control IgG at 4 °C overnight. After six washes, the immunoprecipitated complexes were digested with Proteinase K, followed by RNA extraction. A 10% aliquot of the initial lysate was retained as the input control. All procedures were conducted using the EZ-Magna RIP Kit (17–701, Millipore, United States) according to the manufacturer’s protocol.

### 2.10 Western blotting

BV2 cells and mouse hippocampal tissue were collected for Western blotting analysis. Samples were homogenized in RIPA lysis buffer (P0013C, Beyotime, China) and clarified by centrifugation at 12,000 rpm for 15 min. Supernatant protein concentrations were determined using a BCA assay with reference to a standard curve. Protein samples were resolved on 10% SDS-polyacrylamide gels and electrotransferred onto PVDF membranes. Following blockage with 5% non-fat milk for 1 h at room temperature, membranes were probed overnight at 4 °C with primary antibodies against Sestrin2 (1:1,000; 21,346-1-AP, Proteintech, China), YTHDF1 (1:1,000; 17,479-1-AP, Proteintech, China), and GAPDH (1:3,000; 60,004-1-Ig, Proteintech, China). After washing, the membranes were incubated with HRP-conjugated secondary antibodies (ZB-2305/ZB-2301, Zhongshan Golden Bridge, China) for 1 h. Protein bands were visualized using ECL detection reagent (180–501, Tanon, China) and imaged with a Tanon chemiluminescence imaging system.

### 2.11 Immunocytochemistry

After fixation with 4% paraformaldehyde for 24 h, mouse brains were equilibrated in a 30% sucrose- phosphate-buffered saline (PBS) solution until they sank to the bottom. Brain tissue sections were cut into 10-μm thick slices using a cryostat. To enhance membrane permeability, the sections were treated with 0.3% Triton X-100 in PBS for 15 min, followed by blocking with 10% normal goat serum for 1 h at ambient temperature. Subsequently, the sections were incubated overnight at 4 °C with a rabbit anti-IBA1 primary antibody (1:300; 019–19,741, Wako, Japan). After rinsing, the sections were exposed to Alexa Fluor 594-conjugated anti-rabbit IgG secondary antibody (1:250; A-11012, Invitrogen, United States) for 1 h. Following PBS washes, the section slides were mounted using an anti-fade mounting medium containing DAPI (P0131, Beyotime, China) to label the nuclei. Immunofluorescent images were acquired using an Olympus DP73 microscope.

### 2.12 Statistical analysis

Statistical analyses were conducted using GraphPad Prism 6.0. Comparisons between two groups were evaluated by Student’s *t*-test, while comparisons among multiple groups were assessed using one-way analysis of variance (ANOVA). A significance threshold of *p* < 0.05 was adopted. Each cell-based experiment was repeated at least three times, and all data are expressed as mean ± standard deviation (SD).

## 3 Results

### 3.1 Overexpression of Sestrin2 in microglia alleviates depression-like behaviors

**Figure 1:**
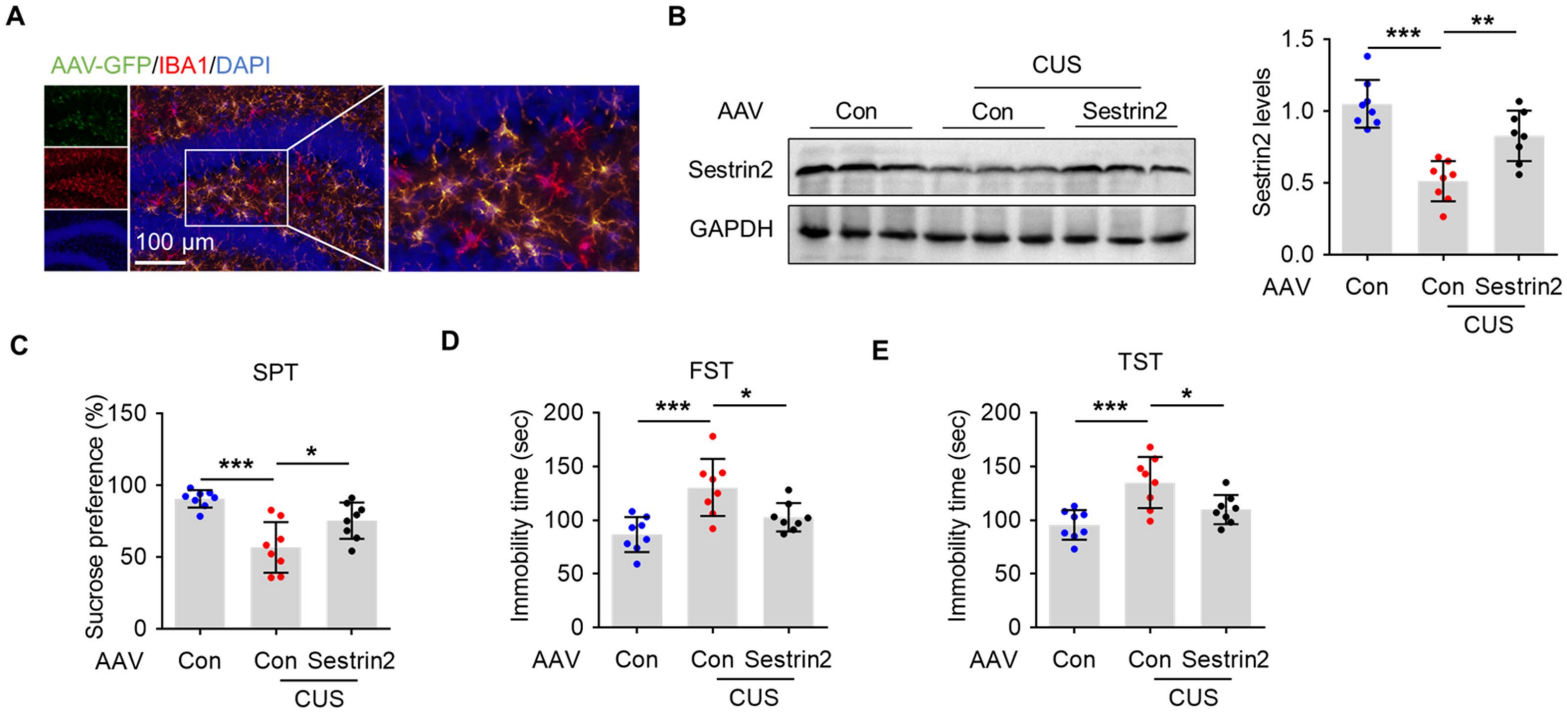
Overexpression of Sestrin2 in microglia alleviates depression-like behaviors. **(A)** Co-localization of GFP fluorescence (green) and IBA1-positive signal (red) in the mouse hippocampus after AAV administration. DAPI staining (blue) was used to label DNA. **(B)** Representative blots showing Sestrin2 expression in the hippocampus of control and CUS mice following microglia-specific Sestrin2 overexpression AAV or vehicle AAV injection. **(C)** Effects of microglia-specific Sestrin2 overexpression AAV or vehicle AAV on sucrose preference values in control and CUS mice. (D-E) Effects of microglia-specific Sestrin2 overexpression AAV or vehicle AAV on immobility time in control and CUS mice during the forced swim test **(D)** and tail suspension test **(E)**. *N* = 8 mice/each group. *p < 0.05, **p < 0.01, and ***p < 0.001.

To investigate the effect of microglial Sestrin2 on depression-like behaviors in mice, we subsequently injected microglia-targeted AAV into the hippocampal region of mice. The results showed the presence of GFP signals originating from the AAV in the mouse hippocampus, and extensive colocalization was observed between the GFP signals and the microglial marker IBA1 signals, indicating that the AAV specifically infected microglial cells (Figure 1A). Sestrin2 expression was significantly reduced in the hippocampal region of CUS mice. Following injection of Sestrin2-overexpressing AAV, the expression level of Sestrin2 in the hippocampus of CUS mice was elevated (Figure 1B). In behavioral tests for depression-related phenotypes in mice, CUS mice exhibited a lower level of sucrose preference, along with significantly increased immobility time in both the forced swim test and the tail suspension test. However, following injection of Sestrin2-overexpressing AAV, the reduced sucrose preference in CUS mice was reversed, and the elevated immobility time in the forced swim and tail suspension tests was suppressed (Figures 1C–E). These results suggest that overexpression of Sestrin2 in microglia alleviates depression-like behaviors in mice.

### 3.2 Overexpression of Sestrin2 in microglia alleviates cognitive impairment and inflammatory response

**Figure 2:**
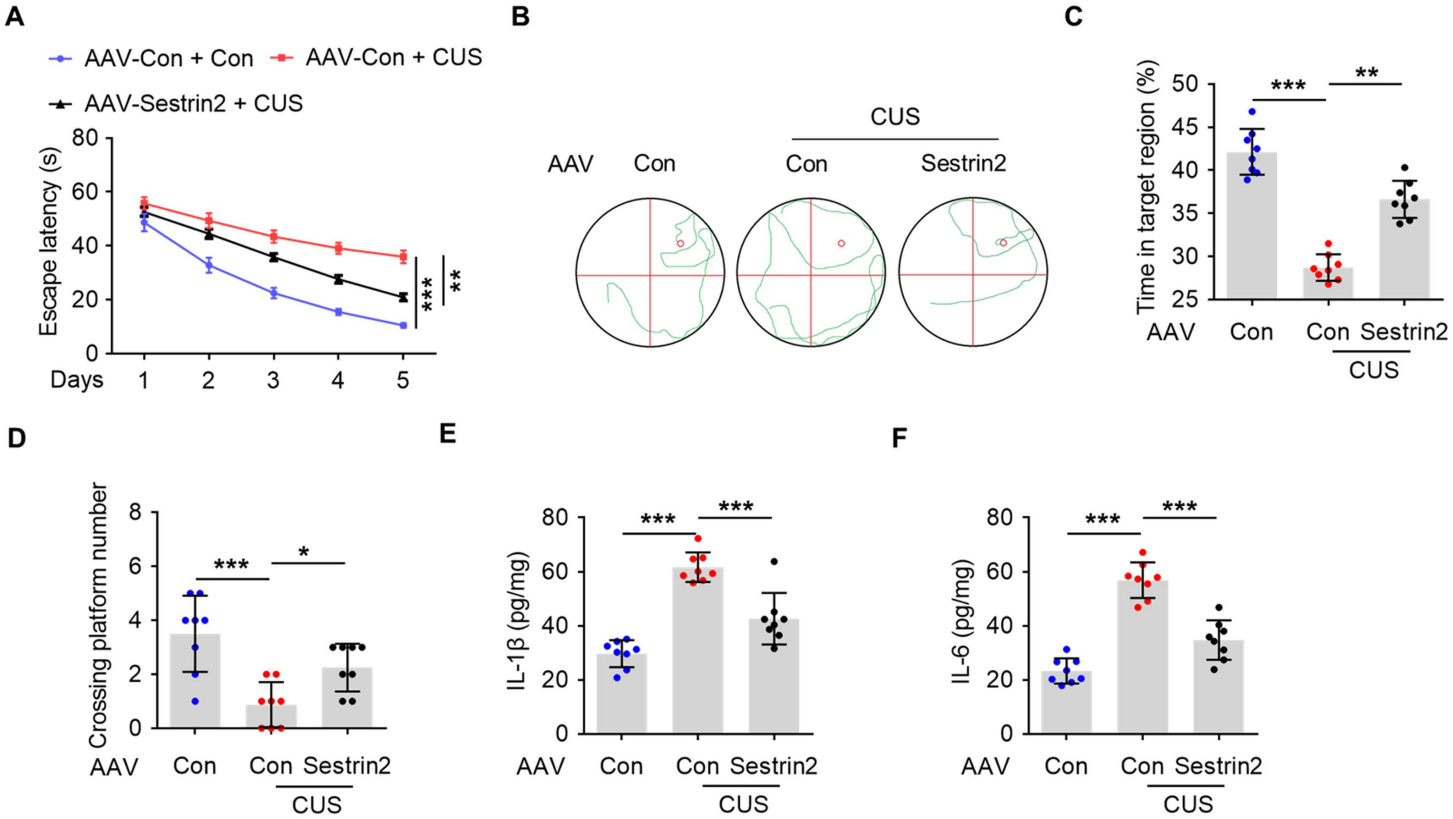
Overexpression of Sestrin2 in microglia alleviates cognitive impairment and inflammatory response. **(A)** The escape latency to get on the hidden platform during the training period in the control and CUS mice injected with microglia-specific Sestrin2 overexpression AAV or vehicle AAV. **(B)** The representative swimming tracks in Morris water maze test of the control and CUS mice injected with microglia-specific Sestrin2 overexpression AAV or vehicle AAV. **(C)** The percentage of time in the target quadrant in the probe test. **(D)** The crossing-platform times in the probe test. **(E,F)** The levels of IL-1β **(E)** and IL-6 **(F)** in the hippocampus of control and DE mice. *N* = 8 mice/each group. *p < 0.05, **p < 0.01, and ***p < 0.001.

To investigate the effect of microglial Sestrin2 on cognitive behavior in mice, we next evaluated cognitive impairment using the Morris water maze. During the training period, the CUS group exhibited a significant decrease in escape latency, whereas Sestrin2 overexpression reversed the reduced escape latency in CUS mice (Figure 2A). During the probe trial, CUS mice spent significantly less time in the target quadrant compared with the control group, and Sestrin2 overexpression reversed the reduced target quadrant time in CUS mice (Figures 2B,C). Similarly, CUS mice showed a lower frequency of platform crossings, whereas Sestrin2 overexpression increased platform crossing frequency in CUS mice (Figure 2D). Furthermore, we examined the inflammatory levels in the hippocampus. The CUS mice exhibited consistently elevated levels of IL-1β and IL-6 in the hippocampus. However, following overexpression of microglial Sestrin2, both IL-1β and IL-6 were significantly reduced (Figures 2E,F). These results suggest that overexpression of Sestrin2 in microglia alleviates cognitive impairment and inflammatory response.

### 3.3 YTHDF1 binds Sestrin2 mRNA in BV2 microglial cells

**Figure 3:**
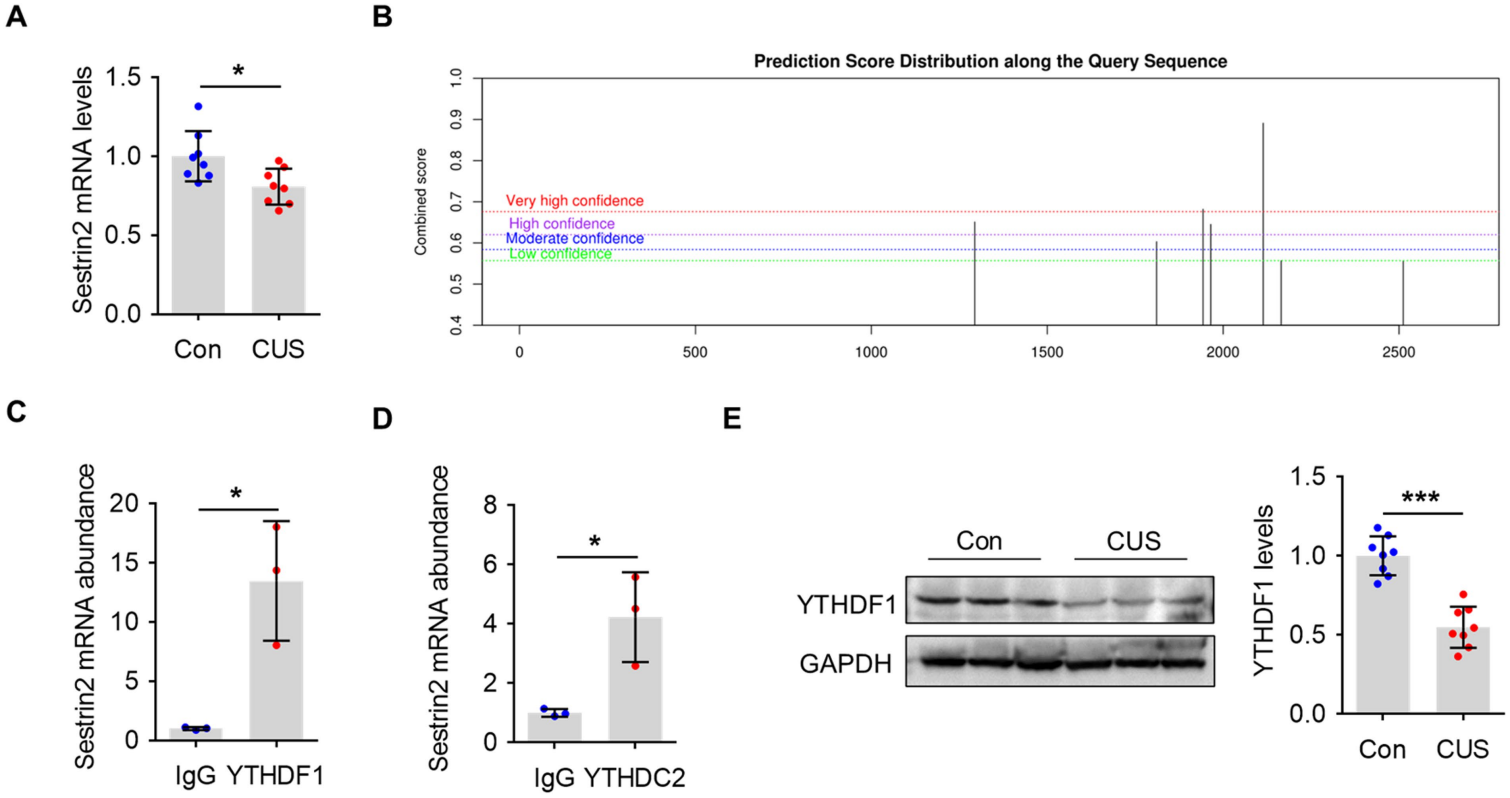
YTHDF1 binds Sestrin2 mRNA in BV2 microglial cells. **(A)** The levels of Sestrin2 mRNA in the control and CUS mouse hippocampus. *N* = 8 mice/each group. **(B)** The predicted schematic of potential m^6^A sites among Sestrin2 mRNA. **(C)** Enrichment levels of Sestrin2 mRNA in BV2 cells following immunoprecipitation with YTHDF1 or IgG antibody. **(D)** Enrichment levels of Sestrin2 mRNA in BV2 cells following immunoprecipitation with YTHDC2 or IgG antibody. **(E)** Representative blots showing YTHDF1 expression in the hippocampus of control and CUS mice. *N* = 8 mice/each group. *p < 0.05 and ***p < 0.001.

To further investigate the mechanism underlying the decreased Sestrin2 expression in the hippocampus of CUS mice, we first examined the level of Sestrin2 mRNA. Sestrin2 mRNA was reduced by approximately 19% in the CUS hippocampus (from 1.0 to 0.807), indicating that while transcriptional suppression exists, post-transcriptional mechanisms likely play a major role in the overall reduction of Sestrin2 expression (Figure 3A). We then analyzed potential m^6^A modification sites within the Sestrin2 mRNA sequence and identified two highly credible sites at positions 1,943 and 2,114 (Figure 3B). Previous studies have indicated that m^6^A modification may enhance protein expression by promoting mRNA translation. YTHDF1 and YTHDC2 are two typical reader proteins that regulate mRNA translation (Zou et al., 2023; Zhang et al., 2024). In the RIP assay, significant enrichment of Sestrin2 mRNA was observed using the YTHDF1 antibody compared with the IgG control group (Figure 3C). In contrast, YTHDC2 failed to yield a comparable enrichment (Figure 3D). Furthermore, we examined YTHDF1 expression in the hippocampus and found that YTHDF1 expression was significantly reduced (Figure 3E). Collectively, these results suggest that microglial YTHDF1 may regulate the translation of Sestrin2 mRNA.

### 3.4 Knockdown of YTHDF1 regulates Sestrin2 via the m6A modified site in Sestrin2 mRNA

**Figure 4:**
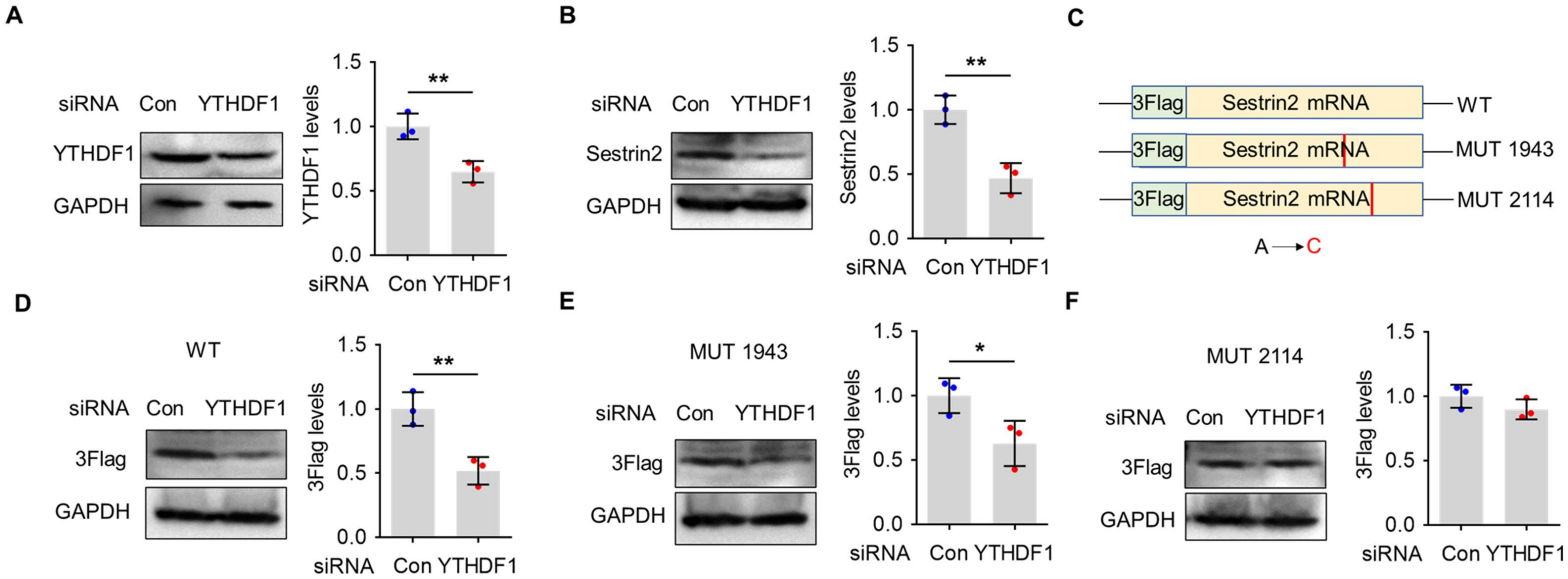
Knockdown of YTHDF1 regulates Sestrin2 via the m^6^A modified site in Sestrin2 mRNA. **(A)** Representative blots showing YTHDF1 expression in the control and YTHDF1 siRNA-transfected BV2 cells. **(B)** Representative blots showing Sestrin2 expression in the control and YTHDF1 siRNA-transfected BV2 cells. **(C)** Schematic diagram of plasmid sequences of wild-type and mutant m^6^A sites of Sestrin2. **(D–F)** Representative blots showing 3Flag expression in the control and YTHDF1 siRNA-transfected BV2 cells pretreated with wildtype **(D)**, MUT1943 **(E)** or MUT2114 **(F)** plasmids. *p < 0.05 and **p < 0.01.

To determine whether YTHDF1 regulates Sestrin2 expression, BV2 cells were transfected with YTHDF1 siRNA, which resulted in suppressed YTHDF1 and Sestrin2 expression levels (Figures 4A,B). Based on the predicted potential m^6^A sites, plasmids carrying site-directed mutations within the Sestrin2 mRNA sequence, each fused with a 3Flag tag, were constructed (Figure 4C). Upon transfection with the wild-type plasmid, YTHDF1 knockdown reduced 3Flag levels (Figure 4D). Similarly, in cells transfected with the plasmid carrying a mutation at position 1943, YTHDF1 knockdown also decreased 3Flag levels (Figure 4E). In contrast, following transfection with the plasmid carrying a mutation at position 2,114, YTHDF1 knockdown did not alter 3Flag levels (Figure 4F). Taken together, these results indicate that YTHDF1 regulates Sestrin2 expression in a manner dependent on the m^6^A site at position 2,114.

### 3.5 Knockdown of YTHDF1 promotes microglial inflammation via Sestrin2

**Figure 5:**
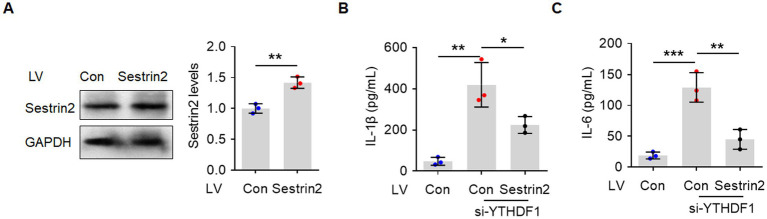
Knockdown of YTHDF1 promotes microglial inflammation via Sestrin2. **(A)** Representative blots showing Sestrin2 expression in the control and Sestrin2 lentivirus-infected BV2 cells. **(B)** The levels of IL-1β in the supernatant of control and Sestrin2-overexpressing BV2 cells following control or YTHDF1 siRNA treatment. **(C)** The levels of IL-6 in the supernatant of control and Sestrin2-overexpressing BV2 cells following control or YTHDF1 siRNA treatment. *p < 0.05, **p < 0.01, and ***p < 0.001.

T To investigate whether YTHDF1 influences the inflammatory response in microglial cells, BV2 cells were infected with Sestrin2-overexpressing lentivirus followed by transfection with YTHDF1 siRNA. Sestrin2-overexpressing lentivirus promoted Sestrin2 expression in BV2 cells (Figure 5A). The results showed that YTHDF1 knockdown led to a marked increase in IL-1β (Figure 5B) and IL-6 (Figure 5C) levels in the cell supernatant. However, in cells overexpressing Sestrin2, the effect of YTHDF1 siRNA was significantly attenuated (Figures 5B,C). Collectively, these data indicate that YTHDF1 regulates the inflammatory response in microglial cells through Sestrin2.

## 4 Discussion

In this study, we identified an important role of microglial Sestrin2 in ameliorating CUS-induced depressive-like behaviors and cognitive impairment. Mechanistically, we demonstrated that YTHDF1 binds to Sestrin2 mRNA in an m^6^A-dependent manner, specifically at the 2,114 modification site, thereby regulating Sestrin2 expression and subsequent microglial inflammatory responses. These findings establish the YTHDF1-Sestrin2 axis as a critical regulator of neuroinflammation in depression and cognitive impairment, which suggests its potential as a novel therapeutic target for MDD.

Sestrin2 has emerged as a stress-inducible protein with pleiotropic functions, including anti-oxidative stress, metabolic regulation, and anti-inflammatory effects (Che et al., 2021; Gao et al., 2020). Numerous clinical studies have revealed substantial associations between Sestrin2 and MDD (Aslan et al., 2023; Su et al., 2024). Moreover, Sestrin2 overexpression attenuates depressive-like behaviors and neuroinflammation in animal models by inhibiting ferroptosis and reducing pro-inflammatory cytokine production (Qian et al., 2023; Ma et al., 2024). Consistent with these findings, our study showed that microglia-specific Sestrin2 overexpression significantly reduced hippocampal inflammation and alleviated behavioral deficits. However, the expression pattern of Sestrin2 appears to be inconsistent across different disease models. In a lipopolysaccharide-induced mouse model of inflammation, Sestrin2 expression was significantly increased in the hippocampus. In cell models, LPS also induced elevated Sestrin2 expression in astrocytes and endothelial cells. Nevertheless, overexpression of Sestrin2 still exerted anti-inflammatory and cytoprotective effects (Pan et al., 2024; Huang et al., 2024). The differential endogenous expression of Sestrin2 in disease states may reflect the body’s homeostatic regulation. Endogenous Sestrin2 expression surges in acute diseases due to the need for tissue repair, whereas it remains at persistently low levels during chronic disease stages, indicative of a pathological state (Wang et al., 2023; Lu et al., 2023; Song et al., 2022). These observations suggest that the direction of Sestrin2 expression change may depend on disease phase and insult type, but its functional role remains predominantly protective.

The m^6^A modification is the most abundant internal modification in eukaryotic mRNA (Hong et al., 2024; Hu et al., 2024). M^6^A reader proteins, especially YTH domain-containing family members, play pivotal roles in post-transcriptional regulation (Jiang et al., 2021; Liao et al., 2022). YTHDF1 is conventionally recognized as a reader that promotes translation of m^6^A-modified mRNAs by recruiting translation initiation factors (Wang et al., 2015). However, recent studies have expanded our understanding of YTHDF1 functions beyond translation regulation. Accumulating evidence indicates that YTHDF1 also participates in mRNA stability regulation, RNA metabolism, and even phase separation-driven formation of membraneless compartments such as P-bodies and stress granules (Ries et al., 2019; Zou et al., 2023). In the nervous system, YTHDF1 has been implicated in synaptic plasticity, learning, and memory formation in the hippocampus (Xu et al., 2022). Furthermore, YTHDF1 has been shown to regulate microglial inflammation in other pathological conditions. A recent study demonstrated that miR-421-3p targets YTHDF1 to inhibit p65 mRNA translation, thereby suppressing inflammatory responses in microglia during cerebral ischemia/reperfusion injury (Zheng et al., 2020). This finding establishes a direct link between YTHDF1 and the regulation of neuroinflammation in microglial cells. Similarly, it’s reported that the WTAP/YTHDF1 axis regulates Lcn2 expression via m^6^A modification, and knockdown of YTHDF1 reduced neuronal death and inflammation in traumatic brain injury (Ma et al., 2024). These findings collectively support the notion that YTHDF1 serves as a crucial regulator of microglial inflammation across various central nervous system disorders.

Our study specifically focused on the role of YTHDF1 in regulating Sestrin2 expression and microglial inflammation. Functional rescue experiments showed that YTHDF1 knockdown promoted IL-1β and IL-6 production in BV2 microglial cells, an effect that was significantly attenuated by Sestrin2 overexpression. The result established that YTHDF1 exerts its anti-inflammatory effects through Sestrin2. This finding is consistent with previous reports demonstrating that YTHDF1 can regulate inflammatory responses by controlling the translation of key inflammatory mediators. For example, YTHDF1 has been shown to promote p65 mRNA translation in an m^6^A-dependent manner, and inhibition of YTHDF1 reduces NF-κB signaling and subsequent pro-inflammatory cytokine production in microglia (Zheng et al., 2020). However, there is a limitation of this study should be acknowledged. While we performed extensive *in vitro* mechanistic studies in BV2 cells, we did not conduct *in vivo* experiments to validate the role of YTHDF1 in animal models. It should be noted that in our RIP assay, YTHDC2 also displayed a modest degree of binding to Sestrin2 mRNA, although its enrichment was markedly lower than that of YTHDF1. While YTHDF1 appears to be the predominant reader protein responsible for regulating Sestrin2 expression in microglia, the possible involvement of other m^6^A readers, including YTHDC2, cannot be entirely excluded. Given that YTHDC2 has been reported to regulate mRNA translation and stability in other cellular contexts, future studies are needed to determine whether YTHDC2 plays a complementary or context-dependent role in modulating Sestrin2 expression and microglial inflammation. Nevertheless, our mutagenesis and functional rescue experiments collectively support a primary role for YTHDF1 in mediating the m^6^A-dependent regulation of Sestrin2. Despite these limitations, our findings and previous animal studies demonstrating the protective role of YTHDF1 in neuroinflammation strongly support our conclusion that the YTHDF1-Sestrin2 axis regulates microglial inflammation and contributes to the pathophysiology of depression.

## 5 Conclusion

Our study demonstrates that microglial Sestrin2 alleviates CUS-induced depressive-like behaviors, cognitive impairment, and neuroinflammation. Mechanistically, YTHDF1 regulates Sestrin2 expression via an m^6^A-dependent mechanism at position 2,114 of Sestrin2 mRNA. The YTHDF1-Sestrin2 axis represents a novel regulatory pathway in depression and a potential therapeutic target for major depressive disorder.

## Data Availability

The original contributions presented in the study are included in the article/supplementary material, further inquiries can be directed to the corresponding authors.
